# District of Columbia Dental Law

**Published:** 1892-07

**Authors:** 


### Editorial, Etc.

DISTRICT OF COLUMBIA DENTAL LAW.—At the meeting of the Maryland State Dental Association in May, Dr. H. B. Noble, of Washington, D. C., in his paper on "Dental Legislation," expressed the hope that before the present session of Congress had passed, a dental law would be enacted for the District of Columbia. His expectations were realized.

The act passed by Congress and approved June 6, 1892 for the regulation of the practice of dentistry in the District of Columbia provides that it shall be unlawful for any person to practice dentistry in the District of Columbia unless such person shall register with the health officer in compliance with with the requirements herein-after provided:

That a board of dental examiners, to consist of five reputable dentists resident of and for three years last before appointment actively engaged in the practice of dentistry in the District of Columbia, is to be appointed by the commissioners of the District for terms of five years and until their successors are appointed; provided, that the first five appointments shall be made for terms of one, two, three, four and five years, respectively. A majority of said board shall constitute a quorum. Vacancies occurring in said board shall be filled by appointment of eligible persons for unexpired terms.

That it shall be the duty of the board of dental examiners to test the fitness and pass upon the qualification of persons desiring to commence the practice of dentistry in said District after the passage of this act and certify to the health officer for registration such as prove, under examination in theory and practice of dentistry, qualified in the judgment of the board to practice dentistry in said District. Provided, that all graduates of dental colleges which require a three years' course of study shall be entitled to certificates upon payment of the certification fee and without examination as to their qualifications.

That it shall be the duty of every person practicing dentistry in said District at the time of the passage of this act to make application to said board, in form prescribed by said board, for certification, and present the certificates thus obtained for registration to the health officer within sixty days from the passage of this act. Every such person so registering may continue to practice without incurring the penalties of this act.

That any one who shall practice or attempt to practice dentistry in the said District without having complied with the provisions of this act shall be deemed guilty of a misdemeanor, and upon conviction thereof shall be fined not less than \$50 nor more than \$200, and in default of payment of such fine shall be imprisoned not less than thirty nor more than ninety days, said fines, when collected, to be paid into the treasury of the United States to the credit of the District of Columbia; provided, that nothing in this act shall be construed to interfere with physicians in the discharge of their professional duties, nor with students pursuing a regular uninterrupted dental college course or in bona fide pupilage with a registered dentist.

That to provide a fund to carry out and enforce the provisions of this act the board of dental examiners may charge such fees, not exceeding \$1 for each certificate and \$10 for each examination, as will from time to time, in the opinion of said board, approved by said commissioners, be necessary. From such fund all expenses shall be paid by the board; provided, that such expenses shall in no case exceed the balance of receipts.

